# Recent progress in metabolic reprogramming in gestational diabetes mellitus: a review

**DOI:** 10.3389/fendo.2023.1284160

**Published:** 2024-01-03

**Authors:** Ya-ping Xie, Shu Lin, Bao-yuan Xie, Hui-fen Zhao

**Affiliations:** ^1^ Nursing Department, The Second Affiliated Hospital of Fujian Medical University, Quanzhou, Fujian, China; ^2^ Centre of Neurological and Metabolic Research, the Second Affiliated Hospital of Fujian Medical University, Quanzhou, Fujian, China; ^3^ Group of Neuroendocrinology, Garvan Institute of Medical Research, Sydney, NSW, Australia

**Keywords:** blood glucose, gestational diabetes mellitus, metabolic reprogramming, pathophysiology, mitochondrial oxidative phosphorylation

## Abstract

Gestational diabetes mellitus is a prevalent metabolic disease that can impact the normal course of pregnancy and delivery, leading to adverse outcomes for both mother and child. Its pathogenesis is complex and involves various factors, such as insulin resistance and β-cell dysfunction. Metabolic reprogramming, which involves mitochondrial oxidative phosphorylation and glycolysis, is crucial for maintaining human metabolic balance and is involved in the pathogenesis and progression of gestational diabetes mellitus. However, research on the link and metabolic pathways between metabolic reprogramming and gestational diabetes mellitus is limited. Therefore, we reviewed the relationship between metabolic reprogramming and gestational diabetes mellitus to provide new therapeutic strategies for maternal health during pregnancy and reduce the risk of developing gestational diabetes mellitus.

## Highlights

- We aimed to provide new therapeutic strategies for maternal health during pregnancy and reduce the risk of developing gestational diabetes mellitus (GDM).
- We investigated the link and metabolic pathways between metabolic reprogramming and GDM.
- Energy metabolism molecules, such as CMPF, miR-143, and SIRT1, may be key regulators and potential therapeutic targets for metabolic reprogramming in GDM; however, the exact mechanisms are unclear.
- Further basic and clinical studies are needed to facilitate the development of new therapies for GDM.

## Introduction

Gestational diabetes mellitus (GDM) is a metabolic abnormality that occurs when women are pregnant and is defined as the first detection of abnormal glucose tolerance in pregnancy (1), accounting for 86.4% of all hyperglycemia cases during pregnancy (2). GDM prevalence is increasing globally due to increasing maternal obesity and age (3). In the last two decades, GDM prevalence in developing countries has increased by over 30% (4). According to the International Diabetes Federation, approximately 14.0% of pregnant women worldwide are affected by GDM (5). It increases the incidence of serious pregnancy outcomes in mothers and infants, such as premature birth, stillbirth, hypertension, and obesity (6, 7). It affects approximately 20 million newborns each year (5).

Patients with GDM are 7 times more likely to develop type 2 diabetes mellitus (T2DM) after delivery compare to healthy women (8), imposing a significant burden on society. However, the pathogenesis and pathophysiological mechanisms of GDM are complex and are not fully understood. Current evidence suggests insulin resistance (IR), β cell dysfunction, and placental dysfunction contribute to its development (9, 10). GDM pathogenesis is associated with abnormal glucose metabolism due to metabolic reprogramming; however, the exact mechanism is unclear (11–13).

Metabolic reprogramming was first observed by Warburg in the 1920s, who discovered that tumor cells could synthesize ATP through glycolysis even under good oxygen conditions, known as the “Warburg effect” (14). Recent technological advancements have enhanced our understanding of metabolic reprogramming. A balanced metabolic system is essential for the structural stability of glucose and normal physiological function. Metabolic processes can adapt to changes in the environment; thus, they can be reprogrammed to support energy requirements in biosynthesis, leading to disease development such as cancer, diabetes, polycystic kidney disease, and vascular inflammatory diseases (15–18). Metabolic reprogramming is also important in GDM development (11–13).

We explored the relationship between GDM and metabolic reprogramming to provide new therapeutic strategies for prenatal care of pregnant women to reduce the risk of developing GDM.

## Metabolic pathways of metabolic reprogramming

Metabolic reprogramming has recently become a popular research area. Metabolic reprogramming refers to the ability of cells to alter their metabolism in order to support the increased energy (19). The main metabolic pathways of metabolic reprogramming are mitochondrial oxidative phosphorylation (OXPHOS) and glycolysis. Metabolic reprogramming leads to the development of many diseases through these processes.

## Mitochondrial OXPHOS

Mitochondrial OXPHOS is a process that occurs in mitochondria, which are important organelles in eukaryotic cells. Mitochondria are involved in many cellular processes, including energy metabolism, oxidative stress regulation, fatty acid oxidation, calcium homeostasis, and the balance between cell survival and death. Given the importance of these processes in the cell, mitochondrial dysfunction may be associated with any disease. Mitochondrial dysfunction results from impaired cellular respiration or defects in mitochondrial quality control, mainly reflected in the OXPHOS machinery (20). Metabolic reprogramming is involved in many diseases, including cancer, diabetes, and rare genetic diseases, mainly through mitochondrial OXPHOS (15, 21, 22). Mitochondrial OXPHOS comprises ATP synthase and the electron transport chain. It regulates the metabolic system by generating membrane potential to drive ATP synthesis and maintain redox homeostasis (20). Mitochondrial ATP synthase, or complex V or F1-FO-ATPase, is the final complex in the OXPHOS cascade reaction. The OXPHOS cascade reaction reduces oxygen by transferring electrons from biological oxidation to the respiratory chain complex and generates an electrochemical proton gradient across the inner membrane. which is used by synthases to generate ATP from inorganic phosphate and ADP (23, 24). The main energy carrier molecule in living organisms is ATP, most of which is produced by mitochondrial ATP synthase via OXPHOS. Mitochondrial OXPHOS can produce ATP at a rate up to 18 times that of aerobic glycolysis (20). OXPHOS dysfunction increases the production of mitochondrial reactive oxygen species clusters, causes oxidative damage and various pathological and aging processes (25).

## Glycolysis

Glucose is the primary carbon and energy source for cells. It provides energy as ATP for metabolites of various biosynthetic pathways (26). Under anoxic conditions, glucose undergoes a series of enzymatic reactions to produce pyruvate, which is then reduced to lactate in a process called anaerobic glycolysis. The extracellular enzyme lactate dehydrogenase (LDH) converts pyruvate to lactate without oxidative phosphorylation. The increase in glycolytic activity ultimately counteracts the effects of hypoxia by generating sufficient ATP from this anaerobic pathway (27). In states where mitochondrial dysfunction occurs in the organism, cells mostly turn to anaerobic glycolysis by increasing the expression of the Lactate Dehydrogenase-Agene(LDHA) (28). Cancer cells exhibit increased glycolysis and glucose uptake rates, generating much ATP and glycolytic intermediates diverted to biosynthetic pathways instead of producing pyruvate (26, 29).

## GDM

**Figure 1:**
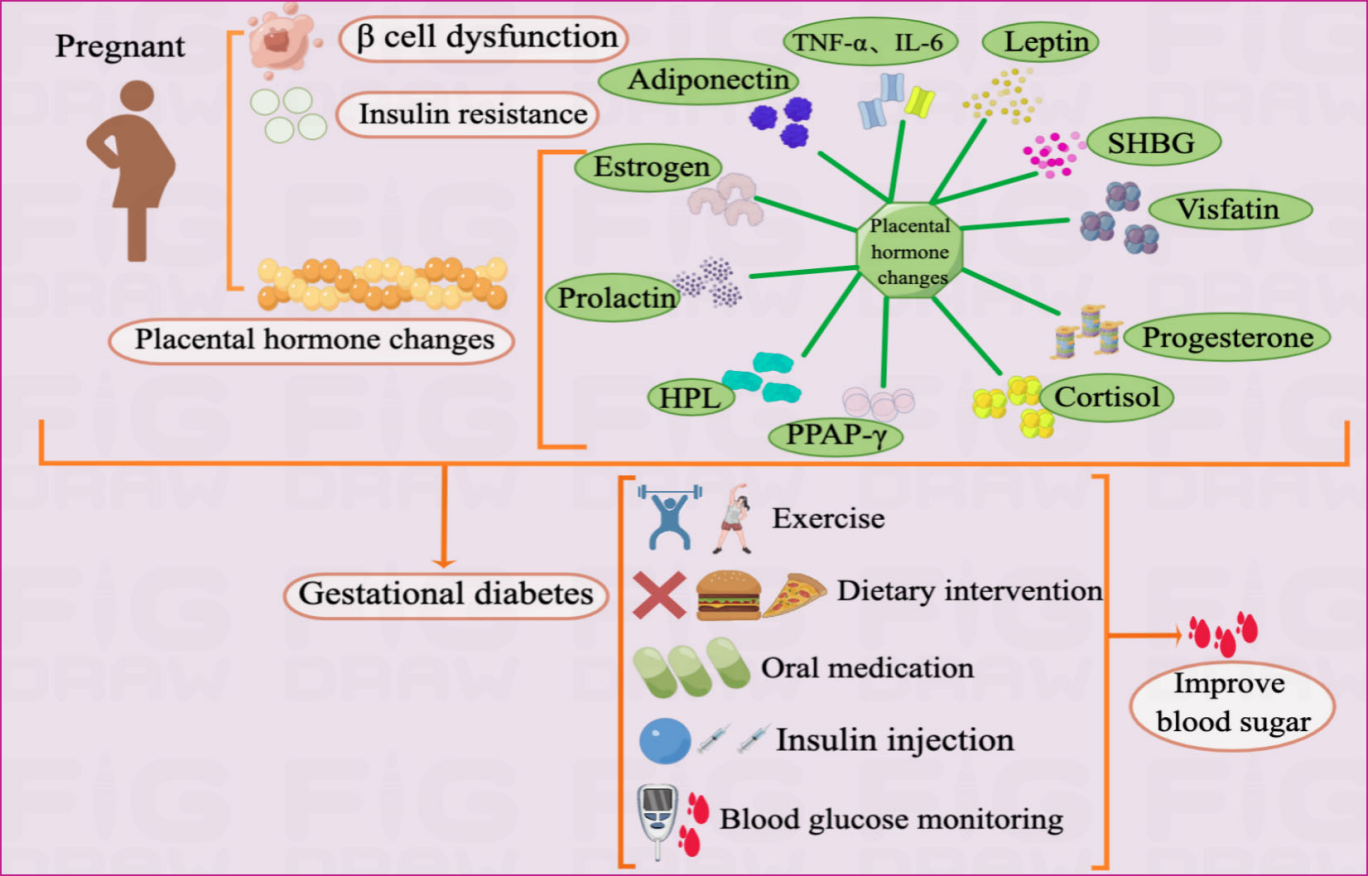
Pathogenesis and treatment of GDM Illustration: The pathogenesis of GDM mainly includes IR, β cell dysfunction, and placental dysfunction. As an endocrine organ, the placenta accelerates inflammatory reactions by secreting adiponectin, leptin, visfatin, progesterone, cortisol, PPAR-γ, HPL, prolactin, SHBG, TNF-α, IL-6 and estrogen, which in turn leads to GDM.The treatment of GDM currently mainly includes drug therapy and non-drug therapy. The drug treatment includes insulin injections and oral medications such as metformin and glyburide. Non-drug treatment mainly includes exercise, diet and self-monitoring of blood sugar.

GDM is mainly caused by metabolic abnormalities during pregnancy, such as IR, β-cell dysfunction, and placental dysfunction (9, 10). Blood glucose can be improved through exercise, diet, drugs, and self-monitoring of blood sugar (**Figure 1**).

## Pathophysiology and pathogenesis

### IR

Maternal obesity, cardiovascular disease, and GDM may be related to IR, which is the reduced efficiency of insulin to promote glucose uptake and utilization. The accumulation of excess lipids or other metabolites can lead to IR, thus, activating inflammatory signaling and endoplasmic reticulum stress (30, 31). Patients with GDM had a 54% reduction in glucose uptake compared to normal pregnancy (32). IR usually results from insulin signaling failure, leading to inadequate GLUT4 membrane transport at the molecular level. And insulin receptor abundance is unaffected; however, decreased levels of insulin receptor tyrosine and increased levels of serine/threonine phosphorylation may inhibit the insulin pathway (33).

**Figure 2:**
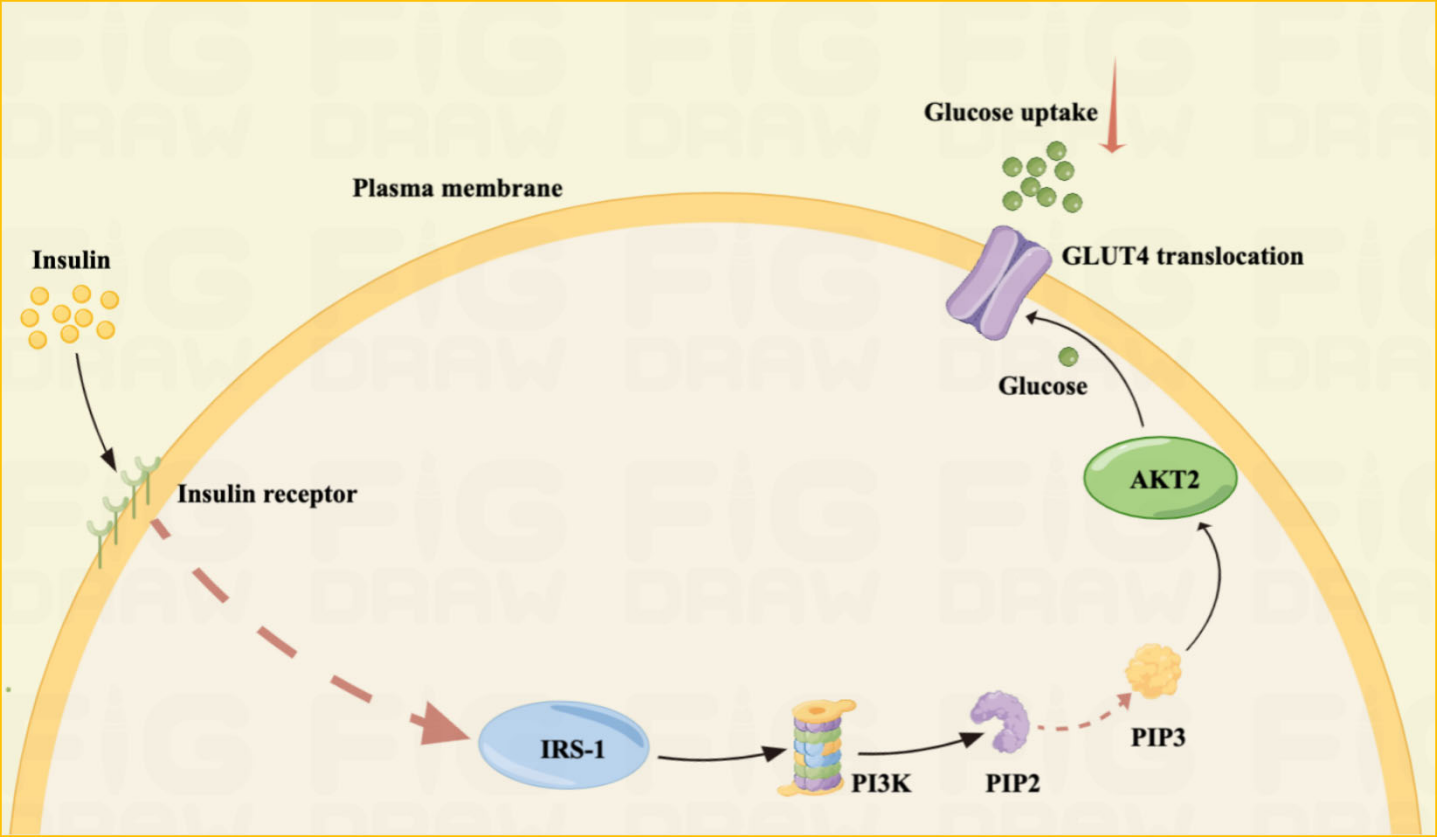
IR mechanism Illustrated: IR is one of the main pathogenesis of GDM.IR is often the result of unsuccessful insulin signaling pathways. Insulin resistance is often the result of unsuccessful insulin signaling pathways. Failure of insulin signaling reduces cellular glucose uptake, leading to GDM in pregnant women. The principle is mainly that insulin activates IRS-1 by binding to the insulin receptor, IRS-1 activates phosphoinositide 3-kinase (PI3K), which further phosphorylates phosphatidylinositol-4,5-bisphosphate (PIP2) into phosphatidylinositol-3,4,5- triphosphate (PIP3). Finally, PIP3 activates Akt2, which facilitates the translocation of GLUT4, allowing glucose uptake into the cell.

Insulin signaling failure reduces cellular glucose uptake, leading to GDM in pregnant women (34). In addition, changes in phosphorylation and/or expression of regulatory factors downstream of the insulin signaling pathway, mainly phosphatidylinositol 3-kinase, GLUT4, and insulin receptor substrate, are the main causes of GDM (32) (**Figure 2**).

## β cell dysfunction

The primary function of β cells is to store and secrete insulin to address glucose load. When β cells fail to detect blood sugar levels adequately or release sufficient insulin is defined as β-cell dysfunction (32). Most of the susceptibility genes associated with GDM may be related to beta cell function, such as glucokinase (Gck) and potassium voltage-gated channel (KQT-like 1, Kcnq1) (34–36). IR exacerbates β cell dysfunction; insulin-stimulated glucose uptake is reduced, leading to the situation of hyperglycemia. This increases the burden on β-cells, forcing them to produce more insulin. Glycotoxicity is the direct effect of glucose on beta cell failure. And, if β cell dysfunction occurs, hyperglycemia, a vicious cycle of IR and more severe β cell dysfunction can be triggered. The number of β-cells is an important regulator of glucose homeostasis (32).

## Placental adipokines and cytokines

As an endocrine organ during pregnancy, the placenta accelerates the inflammatory response by secreting adipokines and cytokines such as adiponectin, leptin, and visfatin, which leads to IR (3, 37) (**Table 1**). Adiponectin, an adipose-derived hormone with anti-inflammatory and insulin-sensitizing effects, may be involved in glucose metabolism during pregnancy (48, 49). It responds to insulin release, helps lower blood glucose levels, and alleviates IR. The mechanism by which adiponectin affects tissues is not fully understood; however, adiponectin binds to its receptor and activates the protein kinase cascade pathway, leading to increased fatty acid oxidation and gluconeogenesis inhibition. In GDM, adiponectin levels are substantially reduced, resulting in IR and hyperglycemia (38). More researchs are needed to explore the role for early prediction of adiponectin levels at 25–28 weeks of pregnancy as a useful biomarker for GDM onset (50, 51). Additionally, adiponectin exerts anti-atherosclerotic and anti-inflammatory effects through the placental barrier and maintains immune interactions between mother and infant blood (51–53).

**Table 1:** Placental adipokines and cytokines.

Factor	Author	Research object	Main conclusion	References
Adiponectin	Akhtar Y et al	Pregnant women from the 24th week to the 40th week of pregnancy	Serum adiponectin concentrations were significantly lower in pregnant women with GDM and adiponectin was inversely correlated with FBG and HbA1c in GDM, suggesting that hypoadiponectinemia is associated with elevated blood glucose during pregnancy.	(38)
Leptin	Roca-rodríguez et al	Pregnant women	GDM participants had significantly higher levels of leptin than controls, therefore, high levels of leptin can be used as a predictive marker for GDM.	(39)
Visfatin	Radzicka-mularczyk et al	Pregnant women	In GDM patients with higher BMI, serum visfatin was elevated, positively correlated with HbA1c, and decreased after insulin treatment.	(40)
Estrogen	Tanaka T et al	C57BL/6 female mice	ERα-mediated estrogen signaling in T cells regulates T cell immunity and contributes to glucose homeostasis during pregnancy.	(10)
PPAP-γ	ZhangY et al	Bewo cells	The PPAR-γ signaling pathway may be involved in the regulation of visfatin by IL-6 in BeWo cells, thereby affecting the pathogenesis of GDM.	(41)
Progesterone	Alyas S et al	Pregnant women with gdm	Significantly elevated progesterone in pregnant women can lead to insulin resistance and eventually GDM.	(42)
HPL	RassieK et al	Women who are pregnant or within 12 months of giving birth	HPL may be valuable as a GDM biomarker, but this meta shows no relationship between HPL and GDM disease status.	(43)
Prolactin	Overgaard M et al	Pregnant women	Low prolactin levels in pregnancy are associated with higher risk of HbA1c and GDM.	(44)
SHBG	Li M et al	Pregnant women	Lower SHBG levels in first trimester were prospectively associated with second trimester insulin resistance and GDM risk. SHBG can be used as a marker to identify high-risk pregnancy in early pregnancy.	(45)
TNF-α、IL-6	Elham H et al	Pregnant women with GDM	Higher circulating levels of IL-6 and TNF-α are associated with increased risk of GDM and can be used as potential biomarkers for the assessment of GDM.	(46)
Cortisol	Sun T et al	24th week pregnant woman	Serum cortisol levels are associated with fasting glucose, triglycerides, and insulin resistance in GDM patients.	(47)

Pregnant women with obesity and GDM have higher leptin concentrations (54). Leptin is an adipocyte-derived hormone controlled by the hypothalamus and is vital in the metabolic feedback regulation of food intake. Abnormal leptin expression can lead to weight gain, irregular food intake, and chronic obesity. This causes the pancreas to secrete more insulin into the bloodstream, a phenomenon known as hyperinsulinemia (51). Higher leptin concentrations can upregulate inflammatory cytokines such as tumor necrosis factor-α and interleukin-6, increasing blood levels and impairing insulin sensitivity, promoting GDM development (41, 55). However, the mechanisms involved remain unclear.

Sex hormone-binding globulin (SHBG) is key in GDM pathogenesis. Disturbances in glucose metabolism cause IR in GDM. Lower SHBG levels in early pregnancy are associated with higher insulin levels, IR, and an increased risk of GDM in mid-pregnancy. Therefore, SHBG can be used to identify high-risk pregnancies in early pregnancy (45, 56). Additionally, SHBG expression negatively correlates with GLUT1 and positively correlates with GLUT3 and GLUT4, suggesting SHBG’s involvement in glucose metabolism by regulating multiple GLUTs, binding to sex hormones or other pathways, thereby eliminating IR in GDM (57). Therefore, SHBG transfection into insulin-resistant cells may be a new approach for GDM treatment.

Visfatin, also known as nicotinamide phosphoribosyltransferase, is an adipocyte cytokine primarily produced by visceral adipose tissue. However, during pregnancy, placental tissue expresses and secretes visfatin (41).Visfatin exerts insulin-like effects via insulin receptor-1 and is associated with IR, inflammation, and obesity. It is involved in the pathogenesis of metabolic disorders such as obesity and GDM (40, 58). Serum visfatin levels are elevated and positively correlated with glycated hemoglobin in patients with GDM with high body mass index but decrease after insulin therapy (40). Furthermore, visfatin-mediated biosynthesis of NAD+ in adipocytes is an important physiological regulator of the metabolic function of the whole body (59).

The expression of substances such as estrogen, progesterone, human placental prolactin, prolactin, and cortisol may change as pregnancy progresses, affecting the peripheral insulin sensitivity of pregnant women (60). This unstable metabolic state increases blood glucose and free fatty acid levels, further contributing to GDM development. However, no consensus exists on whether these hormones contribute to GDM, as some systematic reviews have discovered no association between placental prolactin and prolactin and an increased GDM risk (43, 61). Therefore, further large-scale studies are needed. In GDM, fatty acid binding protein expression is significantly increased in adipocytes due to defective insulin-like growth factor receptor 1 function and phosphorylation of insulin receptors; PPAP-γ expression is decreased, and a chronic inflammatory response occurs (51, 62). PPAR-γ is critical in regulating lipid homeostasis and glucose metabolism in GDM (63). In addition, PPAR-γ ligands upregulate adiponectin expression (41), although the exact mechanism is unknown.

## Current treatment methods and prognosis

GDM can be detected, diagnosed, and treated. Excessive weight gain during pregnancy, pre-pregnancy overweight and obesity, sedentary lifestyle, and maternal age are risk factors for developing GDM (64, 65). GDM management comprises self-monitoring of blood glucose, lifestyle interventions, and medication. Appropriate diabetes management can prevent adverse outcomes and reduce the incidence of adverse maternal and infant outcomes during pregnancy (66). Non-pharmacological interventions are preferred for GDM. Therefore, lifestyle changes are essential for GDM management. The American Diabetes Association (ADA) states that nutritional therapy for GDM can provide enough nutrition to promote health of mothers and their babies, achieve normoglycemia and freedom from ketosis (67). All women with GDM should receive dietary advice from a clinical nutritionist as the basis for non-pharmacological interventions for GDM (68). Dietary recommendations for GDM include sufficient macronutrients and micronutrients to support fetal growth while limiting dietary carbohydrate intake (68). Diet is important in GDM prevention in pregnant women (64). In addition, implementing a carbohydrate-restricted diet or nutritional counseling and other dietary interventions can effectively improve hyperglycemia in people with GDM and delay insulin use during pregnancy (68–70). Exercise interventions, including aerobic and resistance exercise, have also received increasing attention (71). Aerobic or resistance exercise positively affects blood glucose levels, adverse pregnancy outcomes and insulin use in patients with GDM (72–74). More multi-center, large-sample, high-quality RCTs should be conducted to clarify the optimal type, frequency, and duration of exercise and other factors to determine prevention strategies for GDM.

The ADA and the American College of Obstetricians and Gynecologists (ACOG) recommend target glucose levels of <5.3 mmol/l fasting, <7.8 mmol/l 1 hour postprandial, and <6.7 mmol/l 2 hours postprandial (67, 75). For patients with GDM in whom non-pharmacological interventions cannot achieve glycemic control, pharmacological treatment may be initiated if plasma glucose levels exceed target levels by 30%. Insulin is the preferred and only FDA-approved drug (66). Oral medications may sometimes be preferable to insulin injections because they are cheaper, easier to administer, and more acceptable to the patient. Currently, the ACOG recommends oral medications for women who refuse insulin therapy or for whom insulin use is not feasible or safe, with metformin being the preferred oral hypoglycemic agent (76). However, glyburide is an effective treatment for achieving glycemic targets in women with GDM. Clinical experience can minimize the risk of maternal hypoglycemia (77). Glyburide has been widely used in the USA for women with GDM (78). In most guidelines, insulin remains the drug of choice for GDM treatment because the safety and efficacy of other drugs have not been established (67, 79, 80) (**Figure 1**).

## Gestational diabetes and metabolic reprogramming

The relationship between GDM and metabolic reprogramming is poorly understood; however, according to the existing evidence, the metabolic pathways of metabolic reprogramming in GDM may lie mainly in mitochondrial dysfunction, glycolysis, and energy metabolism.

## Gestational diabetes and metabolic reprogramming

### Mitochondrial dysfunction

An interaction exists between high blood glucose concentrations and mitochondrial dysfunction in patients with GDM. Spiral cells isolated from patients with GDM tend to have elevated reactive oxygen species (ROS) levels and vascular dysfunction (11, 13). Glucose is believed to be a source of free radicals, and hyperglycemia promotes lipid peroxidation in low-density lipoprotein through a superoxide-dependent pathway, which is attributed to increased oxidative stress levels in GDM. When hyperglycemia causes free radical production, it disrupts antioxidant defenses. Superoxide dismutase levels are reduced in patients with GDM. Furthermore, hyperglycemia during pregnancy has a detrimental effect on mitochondrial function. Mitochondrial regulatory genes ND2, TFAM, PGC1DNA and NDUFB9 are significantly reduced in patients with GDM (11). Therefore, mitochondrial dysfunction promotes ROS, which may lead to oxidative stress if cellular defense mechanisms are compromised. High levels of oxidative stress, mitochondrial dysfunction, and disrupted antioxidant defense mechanisms occur simultaneously, leading to metabolic damage and cellular changes that lead to GDM development (11).

### Glycolysis and energy metabolism

GDM severely disrupts the metabolism of glucose and fatty acids, suggesting a disturbance in energy metabolism in women with GDM. Compared with normal pregnant women, patients with GDM have significantly lower levels of valine, leucine, lactate, isoleucine, and glycerol-3-phosphate, indicating glycolysis. Glycerol 3-phosphate is synthesized by reducing dihydroxyacetone phosphate with glycerol 3-phosphate dehydrogenase, and its decrease in plasma levels indicates that its production is inhibited. Anaerobic products also indicate glycolysis inhibition in patients with GDM. Impaired absorption and utilization of glucose, resulting in cellular functional reprogramming, and diabetes onset could cause inadequate glucose metabolism in GDM. Triethanolamine is substantially increased in patients with GDM compared with normal pregnant women. Triethanolamine is an important intermediate in glycolysis and acetaldehyde precursor. The substantial increase in triethanolamine may be due to impeded bioconversion of triethanolamine to acetaldehyde, exacerbating energy metabolism disturbance in GDM (12). 2-ketobutyric acid is involved in IR and disrupts glucose homeostasis (81). Plasma 2-hydroxybutyric acid, which produced by 2-ketobutyric acid, concentrations are higher in patients with GDM in late pregnancy than in controls, suggesting that it may be an independent early predictor of glucose abnormalities in humans (82). 3-Hydroxybutyric acid is substantially reduced in patients with GDM, suggesting a disturbance in Krebs’ circulating energy metabolism. Under conditions of insufficient energy, 3-hydroxybutyric acid levels decrease due to its use as a metabolic energy source (83). In addition, pregnant women with obesity are more likely to develop GDM (84). The mechanism is not fully understood; however, this may be due to a high-fat diet (HFD) in patients with obesity leading to hepatic lipogenesis, fat accumulation, lipid oxidation, gluconeogenesis, and glycolysis inhibition. Hepatic glycolytic enzymes PFK-1 and GCK expression is reduced, and PEPCK and G6Pase expression is increased in male mice on HFD. HFD reprograms fuel preference from glucose to fatty acid metabolism, leading to IR (85, 86). This may also be the case in patients with GDM. Glycolysis inhibition and enhanced fatty acid oxidation by HFD may be directly involved in GDM pathogenesis.

Higher glucose levels in the liver cause the separation of lipids from the mitochondrial oxidative pathway, activating serine kinases and inactivating molecules involved in insulin signaling. In fat-overloaded muscles, fatty acid oxidation increases, but downstream Kreb’s energy cycling is blocked, accumulating unprocessed lipid droplets in the mitochondria. This ultimately leads to IR or impaired insulin signaling, resulting in GDM (11).

**Figure 3:**
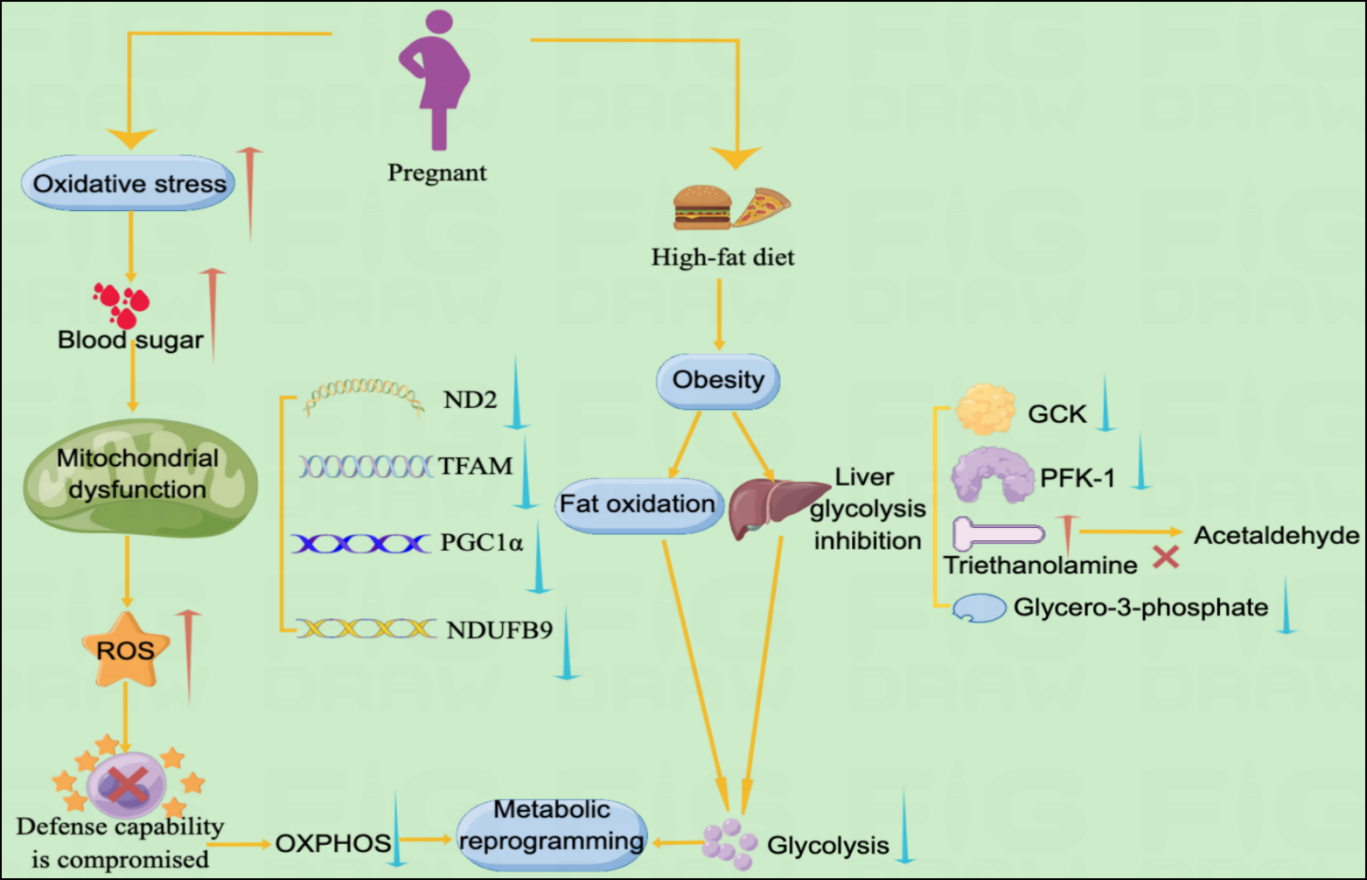
Metabolic pathways of GDM and metabolic reprogramming Illustration: The metabolic pathways of metabolic reprogramming in GDM mainly include mitochondrial dysfunction and the occurrence of glycolysis. Increased levels of oxidative stress in GDM lead to spikes in blood sugar, which further lead to mitochondrial dysfunction. Mitochondrial dysfunction promotes the accumulation of ROS, leading to impaired cellular defense mechanisms, resulting in decreased levels of OXPHOS and ultimately metabolic reprogramming. In addition, obese pregnant women may experience reprogramming of GDM metabolism due to suppressed fat oxidation and decreased levels of glycolysis.

In patients with type 2 diabetes, insulin resistance is induced by changes in OXPHOS levels, reduced NADH oxidoreductase and citrate synthase activity (87). In contrast, data on changes in metabolic reprogramming in patients with GDM are still limited. For women with OXPHOS mutations, the probability of developing GDM is higher (88). It has recently been shown that GDM and hyperglycemia inhibit placental mitochondrial function and glycolysis and alter lipid processing compared to controls. Specifically, GDM had a 39% reduction in ATP content, a 44% reduction in oxidative phosphorylation, and 55%-60% inhibition of CTB glycolysis (89). Although obese pregnant women may disrupt glucose metabolism by inhibiting hepatic glycolysis and fat oxidation, leading to the development of metabolic reprogramming. However, there are still specific data on obese pregnant women or normal weight pregnant women in this regard and more studies are needed to further elucidate this (**Figure 3**).

### The factors involved in metabolic reprogramming in GDM

Many factors regulate GDM metabolic programming. However, most studies have been limited to comparing the level of factors in patients with GDM and normal patients, and less research has been conducted on the underlying mechanisms. Therefore, GDM mechanisms remain unclear (**Table 2**).

**Table 2:** The factors involved in metabolic reprogramming in GDM.

Factor	Author	Research object	Main conclusion	References
NGR 4	Attique H et al	Pregnant women in the 24th to 28th week of pregnancy	NGR4 levels were significantly lower in GDM subjects and may be a potential biomarker of GDM.	(8)
Angptl 8	HuangY et al	Pregnant women in the 12th to 16th week of pregnancy	The level of Angptl8 in early pregnancy is associated with the risk of GDM in pregnancy, which may be used to predict the onset of GDM.	(90)
Smad 4	Li L et al	The htr-8/svneo cells	Deficiency of Smad4 significantly increased GDM insulin sensitivity and attenuated inflammation in insulin-resistant cell models.	(91)
SIRT 1	Ulubasoglu H et al	Pregnant women in the 24th to 28th week of pregnancy	Low SIRT1 levels in the second trimester are associated with GDM. It may be a diagnostic marker for GDM.	(92)
WWOX/HIF1	BarylaI et al	Pregnant women in the 24th to 28th week of pregnancy and 3 months postpartum and 1 year postpartum	The decreased WWOX expression in GDM, especially the decreased WWOX/HIF1A ratio, suggests that WWOX regulates HIF1α activity in normal tissues, gene expression of proteins involved in glycolysis, and may lead to changes in glucose metabolism in GDM.	(93)
HK 2	Song T R et al	Pregnant woman	Upregulation of HK2 can lead to glucose metabolism disturbance in GDM patients.	(94)
Mir-143	Muralimanoharan S et al	Newly delivered mother	Downregulation of miR-143 mediates a metabolic shift from OXPHOS to aerobic glycolysis in GDM placenta.	(95)
CMPF	Yi J et al	Pregnant women in the 24th to 28th week of pregnancy	Elevated serum CMPF levels are not conducive to the occurrence of hyperglycemia and pancreatic β-cell failure in GDM patients, which may affect the occurrence and development of GDM.	(96)

#### Neuregulin 4

Neuregulin 4 (NRG4) is a batokine. It may be important in regulating metabolic homeostasis, insulin sensitivity, and energy maintenance. NRG4 maintains the dynamic balance of glucose and lipids through ERB3 and ERB4 receptors in the liver. It also upregulates GLUT3 and GLUT1 transporters in skeletal muscle to increase glucose uptake and maintain glucose homeostasis (8, 97). However, the exact mechanism of action of NRG4 in GDM is unknown. Patients with GDM have considerably lower NGR4 levels. In contrast, other metabolic factors, particularly the Homeostatic Model Assessment of Insulin and Insulin Resistance, are substantially associated with NGR4. Therefore, NGR4 may be a biomarker for GDM (8). However, larger researchs are needed to explore these results and further determine the potential of NRG4 as a biomarker for GDM.

#### Angiopoietin-like protein 8

Angiopoietin-like protein 8 (Angptl 8) is a newly identified protein factor whose secretion is closely related to lipid metabolism, insulin sensitivity, and glucose metabolism (90). Angptl8 can influence plasma lipoprotein levels by regulating IR and glucose homeostasis (98). JNK signaling pathway inhibition by ANGPTL8 knockdown can enhance insulin sensitivity in trophoblast cells. Thus, ANGPTL8 may be involved in GDM development and provide new insights into its clinical diagnosis and treatment (99). ANGPTL8 levels in early pregnancy are associated with GDM risk (90), which may be used to predict GDM onset and minimize hyperglycemia risk in pregnant women and their offspring.

#### Smad4

Smad4 is a member of the SMAD family of intracellular proteins involved in transducing signals from the transforming growth factor beta (TGF-β) signaling pathway (100, 101). Glucose uptake is widely recognized as the primary source of energy for cellular survival (102). Effective insulin secretion and function are essential for maintaining glucose homeostasis (103). In IR models, Smad4 deficiency impairs insulin sensitivity and suppresses inflammatory responses, whereas Smad4 overexpression has the opposite effect on these changes. TGF-β signals are transmitted to intracellular SMAD proteins, including R-SMADs and Co-SMAD. R-SMAD is phosphorylated at its C-terminal serine residue to form the R-SMAD/Co-SMAD heterodimer. The heteromeric complex is then transported to the nucleus, where it activates or inhibits the transcription of TGF-β responsive genes (91, 104). The main pathological and physiological basis of GDM is manifested as placental IR. Smad4 deficiency substantially increases insulin sensitivity and alleviates the inflammatory response in insulin-resistant cell models. Furthermore, a positive effect of Smad4 on cell viability has been observed (91). The role of Smad4 in regulating IR and inflammatory responses in GDM may confirm its role in metabolic reprogramming and provide new insights into GDM pathogenesis.

#### Sirtuin 1

Sirtuin 1 (SIRT1) is involved in mitochondrial function, energy metabolism, and insulin regulation (105). The T allele of rs3811463 increases SIRT1 protein levels; replacing the T allele with the C allele considerably increases SIRT1 levels. The let-7/Lin28 pathway, which regulates insulin sensitivity by regulating SIRT1 expression (106), is key in regulating glucose and insulin metabolism. Sirt-1 expression is downregulated in patients with GDM (107), and the mechanism of metabolic reprogramming is mainly through oxidative stress, mitochondrial dysfunction, changes in mitochondrial membrane potential, and antioxidant response (108).

#### Hypoxia-inducible factor 1

Hypoxia-inducible factor 1 (HIF1) is encoded by the HIF1a gene and regulates energy metabolism and cellular glucose in pathological processes. Hypoxia is an imbalance in oxygen homeostasis and is essential for metabolism and survival (109). HIF1α protein is crucial in hypoxia. It controls the activity of several target genes involved in metabolism and glucose transport (109). HIF1α upregulation facilitates normal early placental development and embryonic growth as the fetus develops under hypoxic conditions (93, 110). However, in late pregnancy, activation of this protein may lead to poor pregnancy outcomes (111). HIF1α expression is associated with VEGF activation and is linked to placental formation and obesity (112), which may contribute to high HIF1α expression in GDM. WW-domain-containing oxidoreductase (WWOX) regulates glucose metabolism by inhibiting HIF1α in cells and animal models, which affects aerobic glycolysis (93). Skeletal muscle-specific WWOX deficiency disrupts mitochondrial glucose oxidation and stimulates lactate production, disrupting systemic glucose dynamic homeostasis. WWOX deficiency increases HIF1α expression and its target genes, including those that encode important glycolytic enzymes (113). In GDM, WWOX expression is lower than in normal pregnant women and regulates HIF1α activity in tissues. Excessive HIF1α activation leads to the production of glycolysis-related proteins, resulting in pathological changes in glucose metabolism in GDM. Therefore, the regulatory effect of WWOX on HIF1α suggests that the upregulation of glycolytic energy metabolism is important in the metabolic homeostasis of GDM (93). However, data on the effects of gestational diabetes on the WWOX/HIF1 α signaling pathway are limited, and the role of the HIF1α pathway in GDM remains unclear.

#### Hexokinase-2

Hexokinase-2 (HK2) is an enzyme responsible for catalyzing the initial step of most glucose metabolic pathways that involve the conversion of glucose to glucose-6-phosphate by phosphorylation. HK2 is crucial in regulating glycolysis and is overexpressed in different types of cancer, which can alter intracellular glucose homeostasis (114, 115). Reduced glucose-6-phosphate levels in the muscle of patients with IR suggest that abnormal HK2 expression may contribute to IR and diabetes onset. However, the mechanism underlying the correlation between HK2 dysregulation and GDM is unclear. Aerobic glycolysis via HK2 in the placenta of patients with GDM may lead to HK2 expression upregulation due to reduced mitochondrial respiration, resulting in metabolic disorders in patients with GDM. Placental cells rely on glycolysis to facilitate their rapid growth. Primary human trophoblast cells exhibit a higher aerobic glycolysis rate than mature somatic cells. HK1 expression remains stable in the placenta; however, temporary suppression of HK2 may impede glycolytic flow, resulting in feedback inhibition of glucose intake (94). This may explain the reduced glucose uptake observed in HK2-deficient trophoblast cells, although further confirmation is required.

#### MiR-143

MiR-143 has been studied. Extracellular vesicles in the adipose tissue of women with GDM affect glucose metabolism in the placenta. This effect is achieved by increasing the expression of genes related to glycolytic and gluconeogenic pathways (116). Thus, extracellular vesicles can participate in maternal metabolism by regulating adipose tissue activity (117). MiRNA is a small non-coding RNA. It can regulate gene expression by translation inhibition or mRNA degradation (118). MiRNA can regulate cell proliferation, tumorigenesis, and other functions and is involved in cellular physiological or pathological processes, including glucose metabolism (119). Human miR-143 on chromosome 5 inhibits the glycolytic enzyme HK2 and upregulates aerobic glycolysis in cancer cells (95). In GDM, miR-143 downregulation is crucial in glucose metabolism and mitochondrial function, mediating the metabolic transition from OXPHOS to aerobic glycolysis in GDM placentas. In addition, mitochondrial protein expression is reduced in the skeletal muscle of patients with GDM. Therefore, the increased glucose utilization and consumption in GDM placentas may reflect increased expression or activity of limiting enzymes in the glycolytic pathway. Mitochondrial dysfunction and upregulation of glycolytic expression in GDM placentas, including a substantial increase in glycolytic enzyme expression (HK2), phosphofructokinase, and LDH, has been discovered. MiR-143 overexpression partially rescues mitochondrial function, reorganizes mitochondrial respiration, increases the expression of mitochondrial complex proteins, and reduces the expression of glycolytic enzymes by 40% (95). Therefore, miR-143 regulation may be partially responsible for placental mitochondrial dysfunction in GDM. Additionally, miR-143-3p inhibits the Tak1/NF-κB pathway, thereby preventing pancreatic β-cell dysfunction (120).

#### 3-carboxy-4-methyl-5-propyl-2-furanpropanoic acid

3-carboxy-4-methyl-5-propyl-2-furanpropanoic acid (CMPF) is a metabolite of furan fatty acids and is important in glucolipid metabolism. Considerable increases have been observed in women with GDM, postpartum women, patients with impaired glucose tolerance, and patients with T2DM (121, 122). However, the specific role of CMPF in GDM development is unclear. CMPF directly affects pancreatic cells, leading to mitochondrial dysfunction, reduced oxidative stress and glucose-induced ATP accumulation. This may lead to the dysregulation of key transcription factors and a substantial decrease in insulin synthesis levels, resulting in metabolic reprogramming and pancreatic cell dysfunction (121, 123). However, blocking CMPF transport through OAT3 or using antioxidant therapy may prevent CMPF-induced β-cell dysfunction (121). Therefore, CMPF’s role in β-cell dysfunction in GDM/T2DM could be a therapeutic target, and drugs targeting CMPF may improve maternal outcomes and prevent future complications. CMPF may be a biomarker for hyperglycemia and β-cell dysfunction in GDM.

## Conclusions

Metabolic reprogramming in GDM is complex and involves a multifactorial and multistep pathway that can be controlled through mitochondrial OXPHOS and glycolysis. Energy metabolism molecules, such as CMPF, miR-143, and SIRT1, may be key regulators and potential therapeutic targets for metabolic reprogramming in GDM; however, the exact mechanisms are unclear. Owing to the lack of targeted metabolic reprogramming drugs for GDM prevention and treatment, research on GDM can help identify metabolic factors associated with its development and prognosis, which is important to explain the underlying metabolic pathways and improve the diagnosis, treatment, and GDM prognosis. Research can also help to identify novel biomarkers or therapeutic targets. Thus, further basic and clinical studies are needed to facilitate the development of new therapies for GDM.

## Author contributions

Y-PX: Conceptualization, Formal Analysis, Investigation, Writing – original draft, Writing – review & editing. SL: Data curation, Writing – original draft, Writing – review & editing. B-YX: Conceptualization, Writing – original draft, Writing – review & editing. H-FZ: Conceptualization, Supervision, Writing – original draft, Writing – review & editing.
